# Influence of Turkish origin on hematology reference intervals in the German population

**DOI:** 10.1038/s41598-021-00566-2

**Published:** 2021-10-26

**Authors:** Franz X. Mayr, Alexander Bertram, Holger Cario, Michael C. Frühwald, Hans-Jürgen Groß, Arndt Groening, Stefanie Grützner, Thomas Gscheidmeier, Reinhard Hoffmann, Alexander Krebs, Hans-Georg Ruf, Antje Torge, Joachim Woelfle, Oliver Razum, Manfred Rauh, Markus Metzler, Jakob Zierk

**Affiliations:** 1 Department of Pediatrics and Adolescent Medicine, Friedrich-Alexander-University Erlangen-Nürnberg (FAU), Erlangen, Germany; 2 Department of Pediatrics and Adolescent Medicine, University Hospital Erlangen, Erlangen, Germany; 3 MVZ wagnerstibbe, amedes Gruppe, Hannover, Germany; 4 Department of Pediatrics and Adolescent Medicine, University Medical Center Ulm, Ulm, Germany; 5 Paediatric and Adolescent Medicine, Medical Faculty, University Hospital Augsburg, Augsburg, Germany; 6 Core Facility of Clinical Chemistry, University Medical Center Ulm, Ulm, Germany; 7 Institute for Transfusion Medicine and Haemostasis, Medical Faculty, University of Augsburg, Augsburg, Germany; 8 Institute for Laboratory Medicine and Microbiology, Medical Faculty, University of Augsburg, Augsburg, Germany; 9 MVZ Labor PD Dr. Volkmann und Kollegen, Karlsruhe, Germany; 10 Institute of Clinical Chemistry, University Hospital Schleswig-Holstein, Campus Kiel, Kiel, Germany; 11 Department of Epidemiology and International Public Health, School of Public Health, Bielefeld University, Bielefeld, Germany; 12 Center of Medical Information and Communication Technology, University Hospital Erlangen, Erlangen, Germany

**Keywords:** Data mining, Health care, Haematological diseases

## Abstract

Reference intervals for laboratory test results have to be appropriate for the population in which they are used to be clinically useful. While sex and age are established partitioning criteria, patients’ origin also influences laboratory test results, but is not commonly considered when creating or applying reference intervals. In the German population, stratification for ethnicity is rarely performed, and no ethnicity-specific hematology reference intervals have been reported yet. In this retrospective study, we investigated whether specific reference intervals are warranted for the numerically largest group of non-German descent, individuals originating from Turkey. To this end, we analyzed 1,314,754 test results from 167,294 patients from six German centers. Using a name-based algorithm, 1.9% of patients were identified as originating from Turkey, in line with census data and the algorithm’s sensitivity. Reference intervals and their confidence intervals were calculated using an indirect data mining approach, and Turkish and non-Turkish reference limits overlapped completely or partially in nearly all analytes, regardless of age and sex, and only 5/144 (3.5%) subgroups’ reference limits showed no overlap. We therefore conclude that the current practice of using common reference intervals is appropriate and allows correct clinical decision-making in patients originating from Turkey.

## Introduction

Reference intervals (RIs) are used to guide the interpretation of laboratory test results. They provide information on the distribution of test results within a healthy population and are therefore crucial for clinical decision making^1^. In order to increase the sensitivity and specificity of RIs, stratification into subgroups is usually performed, with age and sex being the most common partitioning criteria^1,2^. Although the CLSI guidelines consider ethnic background to be a possible stratification factor and numerous studies have shown the influence of ethnicity on reference limits, most RI studies do not stratify for ethnicity. Likewise, the ethnic background is commonly not taken into account when laboratories adopt reference intervals from manufacturers of in vitro diagnostic devices or from published studies^3^.

Similar to most countries worldwide, the population in Germany is not homogeneous in terms of its ethnic composition. According to the Federal Statistical Office of Germany, 20.8 million people with a so-called migration background lived in Germany in 2018. By the Office’s definition, persons with a migration background either migrated to Germany themselves or are descendants of migrants (irrespective of their current citizenship). They represent a proportion of 25.5% of the total population, and 37.8% of underage persons. Among the persons with a migration background, 35.7% are from European Union countries (including the United Kingdom) and 29.6% originate from European countries outside the EU. About 21.5% are of Asian origin, while the remaining part originates mainly from the African and American continent. Individuals originating from Turkey represent the largest subgroup with about 13.3% of all people with a migration background. In total, about 2.8 million people of Turkish origin lived in Germany in 2018, representing 3.4% of the population^4^.

Previous studies have reported differences in RIs of hematologic parameters between distinct ethnic groups, e.g. RIs for hemoglobin, hematocrit and erythrocyte indices were found to be lower in Black Americans and South- and East Asians than in White Americans living in the United States^3,5^. Moreover, there is ample evidence that RIs for white blood cell count and absolute neutrophil count are lower in the black than in the white population of the United States^6^. However, classification of the Turkish population into these ethnic categories is difficult as the Turkish population’s genetic background is heterogeneous due to major historical migratory movements. Genetic analyses show that the Turkish population has close similarities with both the European and the Middle East populations, and to a certain extent, with South- and Central Asian populations as well^7^.

Analyses of individuals with a migration background in Germany show a different health behavior and health status in comparison to the host population, which can only be partly attributed to socio-economic factors^8–12^. In addition to environmental factors, genetic variation may affect laboratory test results, especially hematologic analytes, in individuals originating from Turkey: Several genetic diseases and traits, which include hemoglobinopathies and enzyme defects, such as alpha and beta thalassemia, sickle cell disease or glucose-6-phosphate dehydrogenase deficiency (G6PDD), lead to changes in hematologic parameters. These conditions have a substantially higher prevalence in Turkey or the Mediterranean region than in Central Europe, and due to migration, these conditions are becoming less regionally concentrated and their prevalence in Germany increases^13^. The prevalence of a genetic predisposition to these diseases in Turkey is estimated at 2.3–10.2% in hemoglobinopathies and up to 18% in G6PDD and is highly dependent on the geographical region^14–16^.

To account for the mentioned genetic and environmental factors’ influence on hematology laboratory test results, geographical origin should be taken into account when establishing reference intervals^3,5,6,17^. However, no peer-reviewed studies have been published so far on hematologic reference intervals for individuals originating from Turkey living in Germany, although this might adversely impact the interpretation of laboratory test results in these individuals.

Different approaches to create reference intervals exist. The conventionally recommended "gold standard" consists of a direct approach in which at least 120 carefully selected healthy reference individuals per group or subgroup are analyzed. The 2.5th and 97.5th percentiles of the distribution of reference values define the lower and upper reference limit^1,2^. However, the creation of RIs using the direct approach is both costly and time-consuming, as it requires defining and recruiting the reference population, obtaining informed consent, sample collection, and analysis. Most importantly, it is limited by ethical objections, especially when pediatric RIs are established, and it is also restricted in geriatric patients due to the high prevalence of comorbidities in this group^18,19^.

An emerging alternative is the so-called indirect approach, in which existing results, e.g. from routine testing, are analyzed in large numbers and appropriate statistical methods are applied to calculate reference intervals. These so-called "data mining" methods have several advantages: Since the data is readily available there is no need for sample collection, bypassing both ethical and logistical challenges and the complex definition of "health(y)”. Additionally, concerns about the procedure of sample acquisition and subsequent analysis are eliminated, as the pre-analytical and analytical conditions are identical to the clinical situation in which the reference intervals are ultimately used. Reference intervals can thus be established in a less resource-intensive way, even for challenging groups like children and elderly patients^2,18,19^.

In this retrospective study we used a validated data-mining algorithm, which estimates RIs from a mixed distribution of pathological and non-pathological test results, and combined it with a validated and data-driven algorithm to determine whether a person is originating from Turkey. Using this approach, we created and compared reference intervals of nine hematologic analytes of the population identified as Turkish or non-Turkish in Germany to assess if differences between them would require stratification by origin.

## Methods

### Analytes

We analyzed nine hematologic parameters (hemoglobin, hematocrit, red cell count, mean red cell hemoglobin, mean red cell hemoglobin concentration, mean red cell volume, red cell distribution width, platelets, and white cell count) as these have a high clinical impact and are among the most frequently performed laboratory tests.

### Study population

In this retrospective study, we gathered data from six German laboratories, of which four are pediatric tertiary care centers and two are laboratory service providers (see Supplemental Table S1). The data was collected between 04/2008 and 08/2017 and contains test results from both outpatients and inpatients. Since not all laboratories contributed data for all analytes, the input datasets for RI calculation of the individual analytes differ. Use of pseudonymized pediatric and adult patient datasets obtained during patient care without patients’ explicit consent is in accordance with the applicable German/Bavarian regulations and has been approved by the Ethical Review Boards of the University Hospital Erlangen, reference number 97_17 Bc.

### Quality analysis and data preparation

Measurements in all laboratories were performed on SYSMEX instruments (detailed information is available in the Supplemental Table S1). A superset of the analyzed dataset has already been evaluated to confirm the comparability of test results between the different centers^20^. Differences between centers’ median values were all within the permitted limits for relative deviation in external quality analysis required by the *“Guideline of the German Medical Association on Quality Assurance in Medical Laboratory Examinations—Rili-BAEK”*^21^*.* To increase between-center homogeneity, we removed the white cell count data from center D based on visual inspection of distribution and median values. The analyte stability over time was also confirmed for each center by analyzing a superset of our data^20^.

In order to minimize the effect of pathological test results on our results and to examine as much data as possible from healthy subjects, we removed data from patients in whom repeated measurements were performed, as we considered the probability of e.g. a severe or chronic disease or a longer hospital stay in these patients to be increased.

**Figure 1:**
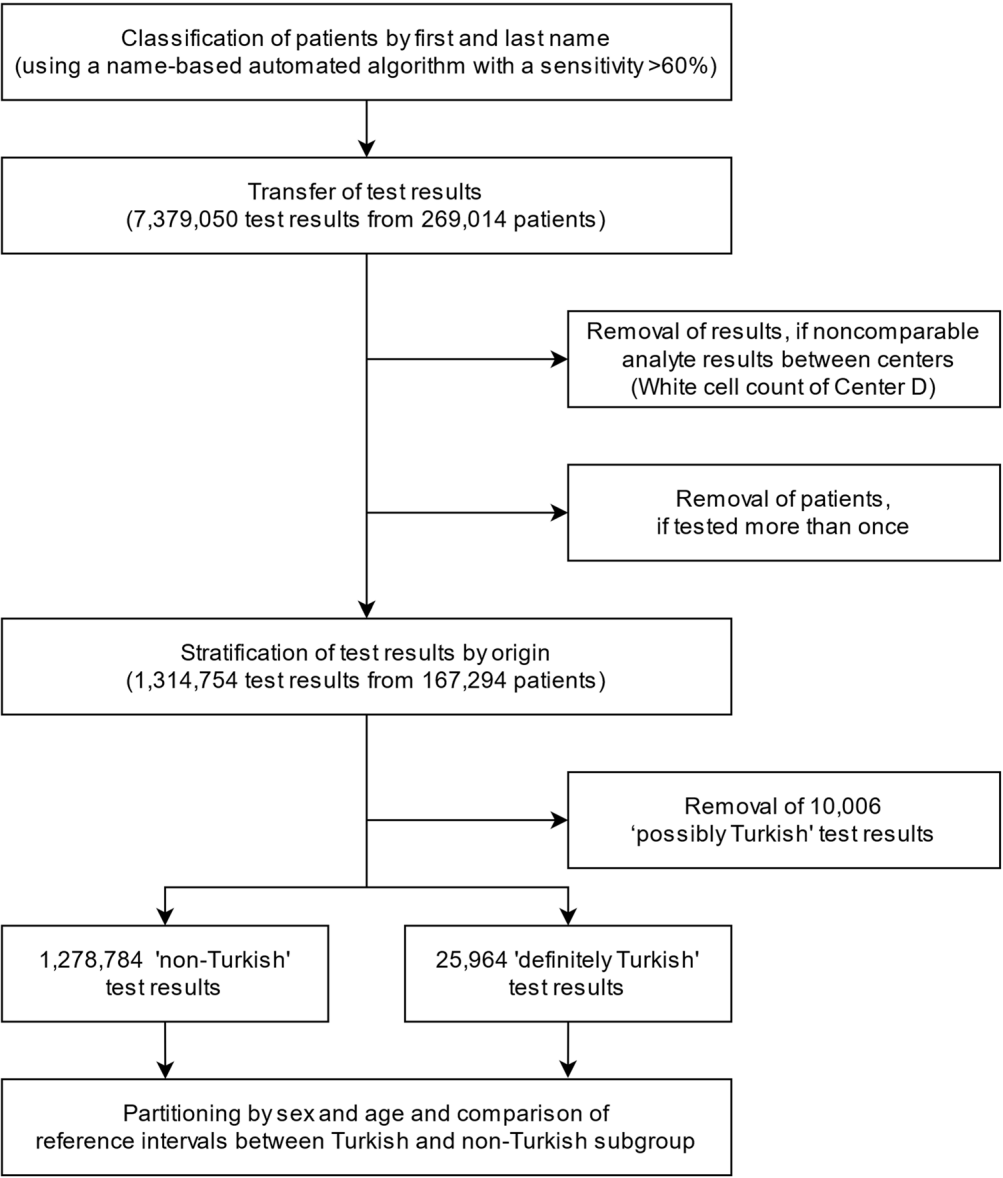
Workflow. Data collection, filtering steps, stratification and calculation of reference and confidence intervals.

A schematic overview of the workflow and the various filtering steps is shown in Fig. 1.

### Identification of Turkish origin using a name-based algorithm

Information concerning a patient’s origin is not recorded in a standardized way in Germany and was therefore not available for analysis. To identify patients with Turkish origin, we used a validated name-based algorithm, which was developed and described by Razum et al. for use in Germany. The background of the method is a name reform in Turkey, which was introduced under former President Kemal Atatürk in 1934 and required every Turkish citizen to adopt and use a family name with a meaning in the Turkish language. Hence, Turkish family names can be distinguished from Central European, Arabic and Asian names and can often be clearly identified as Turkish^22,23^.

The algorithm relies on a list of over 13,000 common Turkish first and last names and consists of an automatic and a manual part. In the automatic part patient names are categorized into "definitely Turkish", "possibly Turkish" or "non-Turkish" based on this list. Individuals with Turkish first and last names, and individuals with a Turkish first or last name and a first or last name which could be of either Turkish or German origin, (a so-called "doublet"), were counted as "definitely Turkish". A classification as "possibly Turkish" was made if only one part of the name was Turkish or a doublet. Accordingly, if no part of the name was Turkish or a doublet it was classified as “non-Turkish”. In the subsequent manual part, the “possibly Turkish” cases were further assessed by a Turkish-speaking researcher based on additional information, like religion or the parents’ names^22–25^. The performance of the algorithm was evaluated in detail in 2008 by Spallek et al. using a manual gold standard. They showed that the automatic part of the algorithm identifies 62.7% of all Turkish cases. If the "possible Turkish" cases are further classified by a human operator using additional information, the sensitivity increases to 97.5%. For both parts, the specificity is 100%, so it can be assumed that the subgroup identified as "definitely Turkish" actually contains only individuals of Turkish origin^24^.

This algorithm has been successfully applied in several studies to identify individuals of Turkish origin in Germany, e.g. to evaluate differences between ethnic groups in cancer incidence rates, cancer treatment response rates or in participation in screening programs^26–28^.

To comply with privacy regulations, name classification was carried out decentralized on laboratory site, and since additional information was not uniformly available, we used the automatic part of the algorithm and subsequently removed the "possibly Turkish" cases from the dataset, thus maintaining the high specificity of the classification.

### Calculation of reference intervals and confidence intervals

To calculate reference intervals and corresponding confidence intervals, we used an indirect data mining approach^29^. The applied *kosmic* algorithm assumes that a dataset obtained as explained above is composed of a major fraction of physiological test results and a minor fraction of pathological test results. It is presumed that the physiological results can be described with a parametric distribution, whereas for the pathological results no specific distribution is assumed. The algorithm uses the Box–Cox-transformation to transform the empirical data into a symmetrical normal distribution and truncates values at both margins. The estimated normal distribution of the remaining central part is used to calculate the 2.5th and 97.5th percentiles, which are defined as the limits of the reference interval. Additionally, the algorithm offers a quantitative measurement of the precision of the reference limits by calculating confidence intervals using a bootstrapping procedure (repeated random sampling with replacement)^29^. The algorithm has been applied in various publications and has proven to provide valid results even if the input dataset contains up to 20–30% pathological test results (e.g. of patients from intensive care units)^20,29,30^. We used a Python binding to the *kosmic* algorithm and calculated the 90% confidence interval for each reference limit with n = 100 bootstrap samples.

### Stratification

As recommended by the CLSI guidelines, we stratified for age and sex^1^. Although age is a continuous parameter, we formed four discrete age groups to ensure a minimum number of test results in each subgroup. This was necessary because the “definitely Turkish” subgroup made up a small fraction of 2.0% of all test results and extensive filtering (removal of all patients with repeat measurements) was performed. Since hematological parameters show distinct dynamics, especially in children and adolescents, we divided pediatric samples into three subgroups (0 to < 6 years, 6 to < 12 years, 12 to < 18 years) and formed an additional age group representing adult patients (≥ 18 years).

## Results

We calculated reference intervals and corresponding 90% confidence intervals for nine hematologic parameters (hemoglobin, hematocrit, red cell count, mean red cell hemoglobin, mean red cell hemoglobin concentration, mean red cell volume, red cell distribution width, platelets, and white cell count) for Turkish and non-Turkish individuals, stratified by sex and age.

In order to examine as many healthy patients as possible and to ensure a reliable classification into "Turkish" and "non-Turkish", we filtered the dataset extensively before applying the algorithm, resulting in a reduction from initially 7,379,050 test results from 269,014 different patients to 1,314,754 test results from 167,294 different patients. About 97.3% of the test results were of “non-Turkish” origin and 2.0% of “definitely Turkish” origin. We excluded the remaining 0.7% test results of “possibly Turkish” origin from subsequent analyses and ultimately examined 24,335–164,523 test results per analyte. Given a reported sensitivity of about 63% of the name algorithm, about 3.1% of the test results in our dataset would therefore in fact be from individuals of Turkish origin. This is reasonably close to data of the Federal Statistical Office, according to which about 3.4% of the total German population have a Turkish migration background^4^.

**Figure 2:**
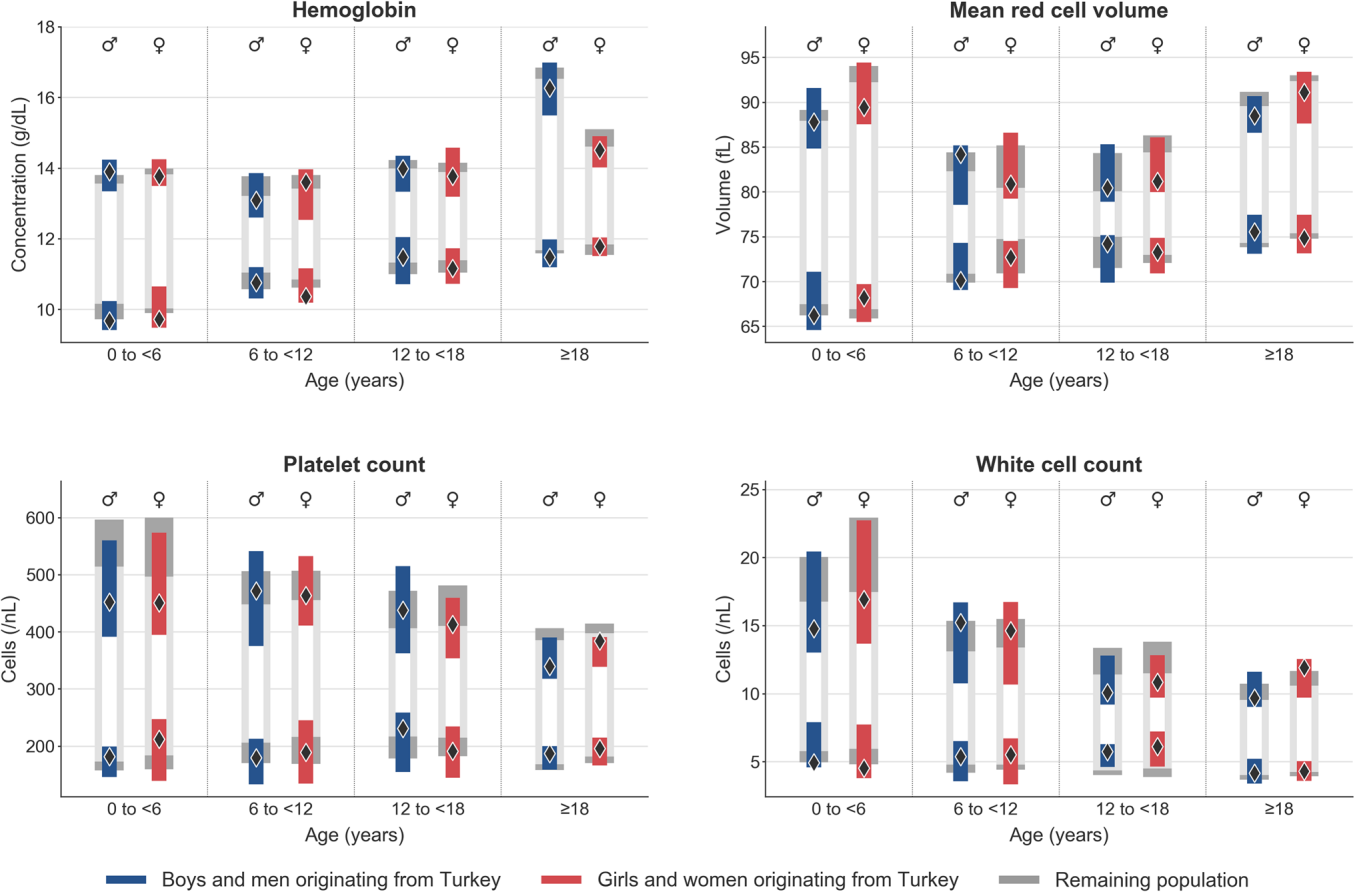
Reference intervals of hemoglobin, mean red cell volume, platelet count, and white cell count for individuals originating from Turkey. Reference intervals and 90% confidence intervals for individuals originating from Turkey (black diamonds denote male and female reference limits and blue and red bars denote the respective 90% confidence intervals) in comparison to the remaining population (dark-gray bars in the background denote reference limits’ confidence intervals). The exact numerical values of the reference intervals and confidence intervals are available in Supplemental Tables S2 and S3. For Red cell count, hematocrit, mean corpuscular hemoglobin (MCH), mean corpuscular hemoglobin concentration (MCHC), and red cell distribution width (RDW) see Supplemental Fig. S1.

The graphical representation of the established reference intervals is shown in Fig. 2 and Supplemental Fig. S1, the exact calculated values including the 50th percentile (median) and the underlying number of test results for the calculation of each subgroup is shown in the Supplemental Tables S2 and S3.

The results show consistently narrower confidence intervals (CIs) for all analytes and age groups in the non-Turkish subgroup compared to the Turkish subgroup due to the larger group sizes in non-Turkish individuals. In the majority of cases, the smaller CI of the non-Turkish subgroup is completely covered by the larger CI of the Turkish subgroup. In a few cases there is an overlap of the confidence intervals. Only in 5/144 (3.5%) reference limits (hematocrit in girls 0 to < 6 years, platelet count in women, mean red cell hemoglobin concentration in boys 0 to < 6 years, and white cell count in boys and girl 12 to < 18 years) completely non-overlapping CIs are observed. Additionally, there are no clearly identifiable trends between the results of the Turkish and non-Turkish subgroups, such as wider or narrower reference intervals or lower or higher reference limits.

## Discussion

We analyzed reference intervals (RIs) and the corresponding confidence intervals (CIs) of nine hematologic laboratory parameters to evaluate if relevant differences between the Turkish and non-Turkish population in Germany would warrant ethnicity-adapted RIs. For this purpose, we combined a name-based algorithm to determine patients’ origin, and an indirect data-mining algorithm to calculate reference intervals.

Our results show that in the majority of RIs, the CIs of the non-Turkish group are within those of the Turkish group or overlap. We therefore conclude that there are no systematic and clinically relevant differences that would warrant separate blood count RIs for individuals originating from Turkey and living in Germany. Although in 5/144 (3.5%) reference limits completely non-overlapping CIs are observed, we do not consider partitioning necessary from a clinical point of view due to only minor differences and lack of systematic differences. The current clinical practice of using common reference intervals is therefore adequate and does not negatively impact clinical decision making in patients originating from Turkey living in Germany.

In order to determine the need for partitioning, we focused on comparing 90% confidence intervals of the reference limits, which is considered the most sensitive method to identify statistically relevant differences between groups. Although several different partitioning criteria for reference intervals have been reported^31–33^, these methods have not been explicitly developed and validated for indirectly derived reference intervals. In general, they are based on analyzing reference values from a healthy reference population, whereas our original dataset consists of both physiological and pathological test results. These methods were therefore not suitable for our approach, although this highlights the need to establish standardized partitioning criteria for indirectly derived reference intervals.

Our study has certain limitations in terms of methodology, number of test results and transferability. Since we filtered the dataset extensively to minimize the effect of diseased patients on our results, the number of test results was reduced to 17.8% of the original dataset. Furthermore, we removed all patients that were classified as “possibly Turkish” resulting in a sensitivity of the aforementioned name-based algorithm of about 63%, while increasing the specificity to 100%, according to previous investigations^24^. However, using this approach, we accepted that more than one third of the individuals of Turkish origin was excluded from subsequent analyses, including all individuals of Turkish origin with a non-Turkish first name or a non-Turkish last name.

Although we identified only about 63% of the individuals of Turkish origin, due to the high specificity we can assume that the individuals identified as originating from Turkey are indeed of Turkish origin, and no 'non-Turkish' individuals influence the results of the Turkish subgroup. Similarly, by removing the 'possibly Turkish' cases from the dataset, we ensured that the influence of individuals with misclassified origin on the 'non-Turkish' group is extremely low with respect to the whole population.

After the filtering steps, the Turkish group accounted for 2.0% of the remaining test results. Considering that about 3.4% of the German population have a Turkish background according to the Federal Statistical Office, the detected 2.0% are close to the expected value of 2.1% for an algorithm with a sensitivity of about 63%. These results are thus consistent and indicate that the name algorithm correctly identifies individuals originating from Turkey in our dataset. As a consequence of further stratification according to sex and age, the number of samples for the RI calculation of the Turkish subgroups was low in some subgroups. The number of test results across all Turkish subgroups ranged from 58 to 1051 and the median was 163. In comparison, the median number of test results in the non-Turkish subgroups was 8289 with a range of 1156–54,215. As expected, this led to narrow CIs within the non-Turkish subgroups and comparatively broad CIs within the Turkish subgroups, which limits the precision and validity of the calculated RIs. In the recommendations on indirectly obtained reference intervals by the IFCC (International Federation of Clinical Chemistry), there is no explicit requirement for a minimum number of underlying test results. The principle "the more, the better" applies and 1000 test results is considered a small number. However, especially for poorly represented groups, smaller numbers are also considered to provide useful information^18^. Although our initial dataset containing 7,379,050 test results already reaches an extraordinary size and acquisition of a larger dataset would be very challenging to realistically achieve, an even larger initial dataset and a higher sensitivity of the name-based algorithm would further improve accuracy.

Our approach to use a name-based algorithm to stratify by geographic origin when establishing reference intervals is novel and has not been reported in the literature so far. For our study, a legal reform in Turkey in 1934 was beneficial, according to which the surnames of Turkish residents had to have a meaning in the Turkish language, increasing the sensitivity of our algorithm^22^. While this approach cannot be applied to all ethnic groups, assigning people to ethnic groups based on their names is not specific to Turkish origin. Name-based algorithms have also been developed for other groups (e.g. South Asians, Chinese, Hispanics) and have been proposed and used for public health and epidemiological questions. Mateos et al. reviewed these algorithms and found high values for sensitivity, ranging from 0.67 to 0.95, and specificity, ranging from 0.8 to 1.0^34^. These algorithms either identify names of a particular group in a dichotomous way or categorize names into multiple ethnic groups or clusters of ethnic groups^34,35^. Recently, Kandt and Longley presented an improved algorithm that allows classification across multiple ethnic groups for individuals in the UK, with an average predictive accuracy of more than 90%^36^. Such algorithms are generally developed by analysing names from electoral registers or census data of a reference population. Limitations in the application to a target population arise the more it differs from the reference population in terms of regional aspects and migration over time. Further limitations exist, for example, in the categorization of persons of mixed origin or of individuals in mixed marriages that assume their partners’ names. Difficulties also emerge when names are common in more than one country or ethnic group^34^. Considering that populations are becoming increasingly diverse due to migration and integration into host societies, it is necessary to highlight that the use of name-based algorithms to identify individuals’ origin will likely become more challenging, as the relationship between individuals’ names and origins becomes less clear.

The optimal use of information regarding patients’ ethnicity (or race) in health care is controversial^37,38^. While the concept of race refers primarily to physical characteristics, such as skin color, or to the geographical origin of ancestors, "ethnicity" is a broader construct encompassing religion, language, common culture, and often common genetic characteristics^37,39^. Population genetics studies have shown substantial differences both within and between racial groups, indicating that a better understanding of variations between different ethnic groups and races can improve patient-based decision-making^37^. In the present study, only a dichotomous classification into "Turkish" and "non-Turkish" origin, based on their names, was made. This simplifies the complex reality of having many different ethnic groups in one country, and ignores the fact that ethnicity is a sophisticated, multidimensional construct that is far more complex than a categorical assignment could represent. However, we considered only the endpoints of a continuous spectrum and could show that there are no relevant differences, indicating that there are no differences within this spectrum either.

While our results do not indicate a need to stratify for patients’ origin in the examined setting (i.e. people of Turkish origin living in Germany), the adoption of origin-specific reference intervals into clinical practice would face several challenges. Unlike age and sex, patients’ origin is not recorded routinely, and its recording is neither standardized nor unequivocal. Thus, patients' origin would have to be determined and categorized, which might raise concerns and lead to practical problems, as self-reported origin or ethnicity is not necessarily a stable characteristic^34^. In addition, laboratory information systems would have to be modified to account for a novel stratification factor for reference intervals.

## Conclusions

We calculated reference intervals (RIs) of hematological parameters to evaluate if there are differences between individuals originating from Turkey and the remaining German population that would necessitate separate RIs. To this end, we used a novel approach by combining a name-based ethnic classification algorithm with an indirect RI calculation method. We demonstrate that for the examined analytes there are either no relevant differences between the studied subpopulations or that from a clinical point of view these differences are not substantial enough to justify separate reference intervals. Thus, we conclude that the current practice of using common reference intervals for Turkish and non-Turkish individuals is appropriate and does not negatively impact clinical decision making in individuals originating from Turkey. In the context of the emerging concepts of personalized medicine, the data mining approach presented here has the potential to improve medical care for other geographically or ethnically defined groups by providing more accurate reference intervals.

## Supplementary Information

Supplementary Information.
