# Supplementary material for: Influence of Turkish origin on hematology reference intervals in the German population
Source: Sci Rep. 2021 Oct 26;11:21074. doi: 10.1038/s41598-021-00566-2 (PMC8548501; doi:10.1038/s41598-021-00566-2)
Supplement: Supplementary file 1 — Supplementary Information. [file 41598_2021_566_MOESM1_ESM.pdf]

Supplemental Material

Franz X. Mayr, Alexander Bertram, Holger Cario, Michael C. Frühwald, Hans-Jürgen Groß, Arndt Groening, Stefanie Grützner, Thomas Gscheidmeier, Reinhard Hoffmann, Alexander Krebs, Hans-Georg Ruf, Antje Torge, Joachim Woelfle, Oliver Razum, Manfred Rauh, Markus Metzler, and Jakob Zierk:

Influence of Turkish origin on Hematology Reference Intervals in the German population

Supplemental Table 1: Participating Centers

Code	Center (all in Germany)	Description and patient population	Analytical device	Patients	Test results
A	University Hospital Erlangen, Erlangen (main center)	Pediatric tertiary care center, all pediatric specialties	SYSMEX XE-2100	51,170	2,662,751
C	University Medical Centre Ulm, Ulm	Pediatric tertiary care center, all pediatric specialties	SYSMEX XE-2100, XE-5000	28,273	1,29,0631
D	Klinikum Augsburg, Augsburg	Pediatric tertiary care center, all pediatric specialties	SYSMEX XN-9000, XP-800i	42,128	1,237,511
E	MVZ Labor PD Dr. Volkmann und Kollegen, Karlsruhe	Private laboratory service provider, mainly primary care samples	SYSMEX XE-2100, XE-2100D	87,094	1,144,906
H	MVZ wagnerstibbe, amedes Gruppe, Hannover	Private laboratory service provider, mainly primary care samples	SYSMEX XN-1000, XN-2000, XT-1800i	18,770	153,120
I	University Hospital Schleswig-Holstein, Kiel	Pediatric tertiary care center, all pediatric specialties	SYSMEX XN, XS-800i, XE-2100, XT-1800i, XN-350	41,579	890,131

Supplemental Table 2: Male results

PARAMETER	UNIT	AGE GROUP (years)	Non-Turkish										Turkish									
				Lower RL (2.5th Percentile)			Median (50th Percentile)			Upper RL (97.5th Percentile)				Lower RL (2.5th Percentile)			Median (50th Percentile)			Upper RL (97.5th Percentile)		
			N	VALUE	90% CI		VALUE	90% CI		VALUE	90% CI		N	VALUE	90% CI		VALUE	90% CI		VALUE	90% CI	
**HGB**	g/dL	0 to <6	15384	9,8	9,7	10,2	11,7	11,7	11,8	13,7	13,6	13,8	344	9,7	9,4	10,2	11,6	11,4	11,9	13,9	13,4	14,2
		6 to <12	8268	10,6	10,6	11,0	12,1	12,1	12,2	13,6	13,2	13,8	144	10,8	10,3	11,2	11,9	11,8	12,1	13,1	12,6	13,8
		12 to <18	7368	11,0	11,0	11,3	12,6	12,6	12,6	14,2	14,0	14,2	156	11,5	10,7	12,0	12,7	12,3	12,8	14,0	13,3	14,3
		≥18	47354	11,6	11,6	11,7	13,9	13,9	14,0	16,7	16,5	16,8	861	11,5	11,2	12,0	13,7	13,5	13,9	16,3	15,5	17,0
**HCT**	%	0 to <6	15395	29,3	29,1	29,5	34,6	34,5	34,7	40,8	40,6	41,1	338	29,4	28,3	30,6	34,8	34,4	35,6	41,2	39,7	42,6
		6 to <12	8288	31,6	30,9	32,2	35,3	35,2	35,5	39,2	38,6	40,3	150	31,1	30,4	32,9	35,7	34,8	36,3	41,0	37,4	41,5
		12 to <18	7383	32,0	31,9	32,7	36,5	36,4	36,6	41,1	40,5	41,5	155	33,2	32,1	34,9	37,2	36,6	37,7	41,4	38,9	42,3
		≥18	47475	33,7	33,5	33,8	40,8	40,6	40,9	49,3	48,8	49,6	859	33,6	32,7	34,4	40,0	39,5	40,9	47,6	45,6	50,4
**RBC**	/pL	0 to <6	15960	3,87	3,77	3,91	4,61	4,56	4,61	5,48	5,28	5,52	343	3,86	3,72	4,10	4,63	4,52	4,70	5,45	5,18	5,66
		6 to <12	8401	4,00	3,96	4,11	4,59	4,56	4,60	5,27	5,08	5,28	154	4,00	3,90	4,17	4,60	4,51	4,68	5,28	4,91	5,40
		12 to <18	7517	4,05	4,04	4,17	4,65	4,64	4,68	5,25	5,18	5,33	154	4,18	3,86	4,44	4,77	4,66	4,86	5,39	5,07	5,63
		≥18	48109	4,31	4,23	4,38	4,95	4,93	4,96	5,64	5,57	5,77	843	4,18	4,20	4,49	4,97	4,91	5,05	5,78	5,54	5,83
**MCH**	pg	0 to <6	15391	22,6	22,5	22,7	26,3	26,2	26,3	30,6	30,4	30,7	343	23,0	21,9	24,2	26,0	25,8	26,3	29,2	28,3	31,0
		6 to <12	8273	24,4	24,2	24,9	26,7	26,5	26,7	29,1	28,3	29,1	160	25,0	24,1	25,4	26,2	26,0	26,4	27,4	27,0	28,4
		12 to <18	7381	25,3	24,8	25,5	27,1	27,0	27,2	28,9	28,6	29,5	162	24,9	23,6	25,4	26,9	26,2	27,1	28,8	28,3	29,6
		≥18	47492	25,8	25,7	25,9	28,5	28,4	28,5	31,5	31,0	31,6	836	25,3	25,0	25,9	28,2	27,8	28,4	31,4	30,4	31,6
**MCHC**	g/dL	0 to <6	15402	31,1	30,9	31,3	34,0	34,0	34,1	37,2	36,9	37,3	342	31,1	30,7	31,6	33,3	33,1	33,8	35,7	34,9	36,7
		6 to <12	8289	32,1	31,9	32,5	34,4	34,3	34,5	36,8	36,1	36,8	158	31,9	31,3	32,8	33,6	33,5	33,9	35,3	34,6	36,4
		12 to <18	7387	32,5	32,4	32,7	34,6	34,6	34,7	36,8	36,5	36,8	161	33,0	31,5	33,4	34,5	34,0	34,5	36,0	34,9	36,8
		≥18	47495	32,9	32,7	32,9	34,7	34,7	34,7	36,7	36,5	36,7	859	32,6	32,2	33,2	34,5	34,2	34,6	36,6	35,5	36,8
**MCV**	fL	0 to <6	15975	67	66	67	77	77	77	89	88	89	335	66	65	71	77	76	78	88	85	91
		6 to <12	8400	70	70	71	77	76	77	84	82	84	163	70	69	74	77	76	78	84	79	85
		12 to <18	7531	75	72	75	78	77	78	80	80	84	152	74	70	75	77	77	78	80	79	85
		≥18	48165	74	74	74	82	82	82	91	90	91	861	76	73	77	82	81	82	88	87	91
**RDW**	%	0 to <6	3567	10,1	9,7	11,5	15,3	15,1	15,7	20,4	19,9	21,0	183	9,5	8,7	12,4	14,7	14,2	15,7	20,3	17,3	21,3
		6 to <12	1438	12,2	12,1	12,3	13,5	13,4	13,6	14,9	14,6	15,2	69	11,8	9,9	13,1	13,5	12,4	14,0	15,4	14,2	16,0
		12 to <18	1292	12,1	12,0	12,4	13,2	13,1	13,3	14,5	14,1	14,6	77	11,8	11,6	12,9	13,5	13,1	13,9	15,5	14,2	15,8
		≥18	5651	11,9	11,9	12,0	12,9	12,9	13,0	14,0	13,8	14,1	321	12,1	11,8	12,3	13,0	12,9	13,2	14,0	13,8	14,6
**PLT**	/nL	0 to <6	15963	168	157	173	315	304	317	590	514	596	338	182	147	198	286	274	306	452	393	559
		6 to <12	8414	194	170	205	316	308	319	497	448	506	153	179	134	211	293	276	321	472	376	539
		12 to <18	7537	189	178	217	302	298	311	434	406	472	149	230	156	257	318	297	336	438	363	513
		≥18	48168	162	158	168	260	258	261	401	385	406	837	187	160	198	255	250	262	339	319	389
**WBC**	/nL	0 to <6	13041	5,5	4,9	5,8	10,2	9,9	10,4	18,7	16,8	20,0	310	4,9	4,6	7,8	9,8	9,8	11,3	14,8	13,1	20,4
		6 to <12	7076	4,6	4,2	4,8	8,2	7,9	8,3	14,6	13,1	15,3	131	5,4	3,6	6,5	9,1	8,2	9,5	15,2	10,8	16,6
		12 to <18	6476	4,3	4,0	4,4	7,3	7,1	7,5	12,4	11,4	13,4	145	5,7	4,7	6,2	7,6	7,4	8,1	10,1	9,2	12,7
		≥18	41540	3,8	3,7	4,0	6,3	6,3	6,5	9,9	9,5	10,7	744	4,2	3,4	5,2	6,9	6,7	7,2	9,7	9,1	11,6

Supplemental Table 3: Female results

PARAMETER	UNIT	AGE GROUP (years)	Non-Turkish										Turkish									
				Lower RL (2.5th Percentile)			Median (50th Percentile)			Upper RL (97.5th Percentile)				Lower RL (2.5th Percentile)			Median (50th Percentile)			Upper RL (97.5th Percentile)		
			N	VALUE	90% CI		VALUE	90% CI		VALUE	90% CI		N	VALUE	90% CI		VALUE	90% CI		VALUE	90% CI	
**HGB**	g/dL	0 to <6	13416	10,0	9,9	10,0	11,8	11,7	11,8	13,9	13,8	14,0	323	9,7	9,5	10,6	11,7	11,6	12,1	13,8	13,5	14,2
		6 to <12	6660	10,7	10,6	10,8	12,2	12,1	12,2	13,8	13,4	13,8	163	10,4	10,2	11,1	11,9	11,7	12,2	13,6	12,6	13,9
		12 to <18	6226	11,1	11,0	11,4	12,6	12,6	12,6	14,0	13,9	14,2	135	11,2	10,7	11,7	12,4	12,3	12,7	13,8	13,2	14,6
		≥18	53424	11,7	11,5	11,8	13,2	13,2	13,3	14,7	14,6	15,1	994	11,8	11,5	12,0	13,1	13,0	13,2	14,5	14,0	14,9
**HCT**	%	0 to <6	13428	29,3	29,1	29,5	34,8	34,8	34,9	41,4	41,2	41,6	335	31,9	29,6	32,9	36,1	35,3	36,6	40,5	39,7	42,1
		6 to <12	6667	31,4	31,2	31,8	35,5	35,3	35,7	40,1	39,3	40,6	158	32,4	30,0	33,9	36,0	35,1	36,4	39,5	38,3	41,8
		12 to <18	6242	32,2	31,8	32,5	36,8	36,6	36,9	41,9	41,1	42,0	132	32,9	32,1	35,4	37,3	36,9	38,0	41,8	39,7	42,8
		≥18	53501	34,4	34,1	34,7	38,9	38,8	39,0	43,9	43,5	44,2	1018	34,3	34,3	35,8	39,0	38,7	39,4	43,7	42,7	44,1
**RBC**	/pL	0 to <6	13940	3,77	3,71	3,87	4,54	4,51	4,55	5,43	5,21	5,48	328	4,01	3,66	4,18	4,67	4,54	4,73	5,45	5,22	5,58
		6 to <12	6788	4,00	3,95	4,10	4,57	4,56	4,60	5,15	5,11	5,20	160	4,22	3,87	4,37	4,74	4,56	4,78	5,26	4,94	5,49
		12 to <18	6324	4,03	4,00	4,10	4,63	4,60	4,65	5,30	5,18	5,31	135	4,01	3,91	4,30	4,69	4,61	4,78	5,42	5,06	5,50
		≥18	54148	4,02	4,00	4,09	4,63	4,63	4,65	5,25	5,24	5,30	1007	4,25	4,04	4,35	4,65	4,63	4,71	5,05	4,97	5,34
**MCH**	pg	0 to <6	13426	23,3	22,6	23,3	27,0	26,8	27,0	31,2	31,1	31,7	331	23,0	22,1	24,4	26,6	26,3	27,3	30,9	29,8	32,4
		6 to <12	6665	24,8	24,6	25,1	26,8	26,8	26,9	28,9	28,6	29,1	162	24,9	23,9	25,5	26,3	26,1	26,6	27,8	27,3	29,0
		12 to <18	6252	25,2	25,0	25,7	27,4	27,2	27,4	29,5	28,9	29,7	129	25,5	23,8	26,2	27,4	26,8	27,6	29,5	28,1	30,9
		≥18	53521	25,9	25,8	26,2	28,7	28,6	28,7	31,5	31,2	31,6	1010	26,2	25,4	26,8	28,1	28,0	28,4	30,1	29,6	31,3
**MCHC**	g/dL	0 to <6	13437	31,1	30,9	31,5	34,0	33,9	34,0	37,1	36,4	37,1	328	30,8	30,5	31,6	33,4	33,1	33,8	36,3	34,9	36,7
		6 to <12	6665	32,0	31,8	32,4	34,2	34,1	34,3	36,5	36,0	36,6	159	32,6	31,9	33,0	33,8	33,6	34,0	35,0	34,4	36,1
		12 to <18	6249	32,3	32,2	32,6	34,4	34,3	34,5	36,5	36,3	36,6	131	31,3	30,9	32,4	33,5	33,3	34,3	35,9	35,0	37,2
		≥18	53571	32,1	32,0	32,4	34,1	34,1	34,2	36,2	36,1	36,2	992	31,7	31,3	32,2	33,8	33,7	34,0	35,9	35,7	36,3
**MCV**	fL	0 to <6	13953	66	66	67	79	78	79	94	92	94	330	68	66	70	78	77	79	89	88	94
		6 to <12	6794	72	71	75	78	77	78	85	80	85	155	73	69	74	77	76	78	81	79	87
		12 to <18	6332	72	72	73	79	78	79	86	84	86	137	73	71	75	77	77	79	81	80	86
		≥18	54215	75	75	75	84	84	84	93	92	93	1025	75	73	77	83	82	83	91	88	93
**RDW**	%	0 to <6	3409	12,8	11,7	13,1	16,3	16,0	16,4	19,7	19,6	20,4	150	11,7	10,1	12,6	14,0	13,7	15,0	16,7	15,5	20,4
		6 to <12	1176	12,5	12,0	12,8	13,4	13,3	13,4	14,2	14,1	14,8	70	11,7	10,5	13,1	13,2	12,9	13,7	14,8	14,1	16,0
		12 to <18	1156	12,1	11,9	12,4	13,0	12,9	13,1	13,8	13,6	14,2	58	12,6	11,6	12,8	13,2	12,9	13,4	13,8	13,5	14,7
		≥18	5395	11,9	11,8	12,0	12,8	12,7	12,8	13,6	13,5	13,8	323	12,2	11,6	12,4	12,9	12,8	13,1	13,7	13,5	14,4
**PLT**	/nL	0 to <6	13938	173	159	184	314	306	321	563	496	599	337	212	140	246	318	292	325	451	396	572
		6 to <12	6791	174	168	216	314	313	328	468	455	507	156	189	135	243	326	296	335	464	412	531
		12 to <18	6338	202	182	215	312	301	315	482	410	481	130	191	145	233	291	274	306	413	355	458
		≥18	54134	177	170	181	273	271	274	410	397	414	1051	196	167	213	276	267	281	384	340	389
**WBC**	/nL	0 to <6	11553	5,7	4,8	5,9	10,8	10,4	11,0	20,0	17,4	22,9	272	4,5	3,8	7,7	10,2	9,9	11,4	16,9	13,7	22,7
		6 to <12	5757	4,6	4,4	4,8	8,2	8,0	8,3	14,5	13,4	15,5	146	5,5	3,4	6,6	9,0	8,3	9,4	14,6	10,7	16,7
		12 to <18	5514	4,2	3,9	4,5	7,5	7,2	7,6	13,2	11,5	13,8	127	6,1	4,7	7,1	8,4	8,1	8,9	10,8	9,8	12,8
		≥18	47533	4,0	3,9	4,2	6,7	6,7	6,8	11,4	10,6	11,7	877	4,3	3,6	5,0	7,2	6,7	7,3	11,9	9,8	12,5

Supplemental Figure 1

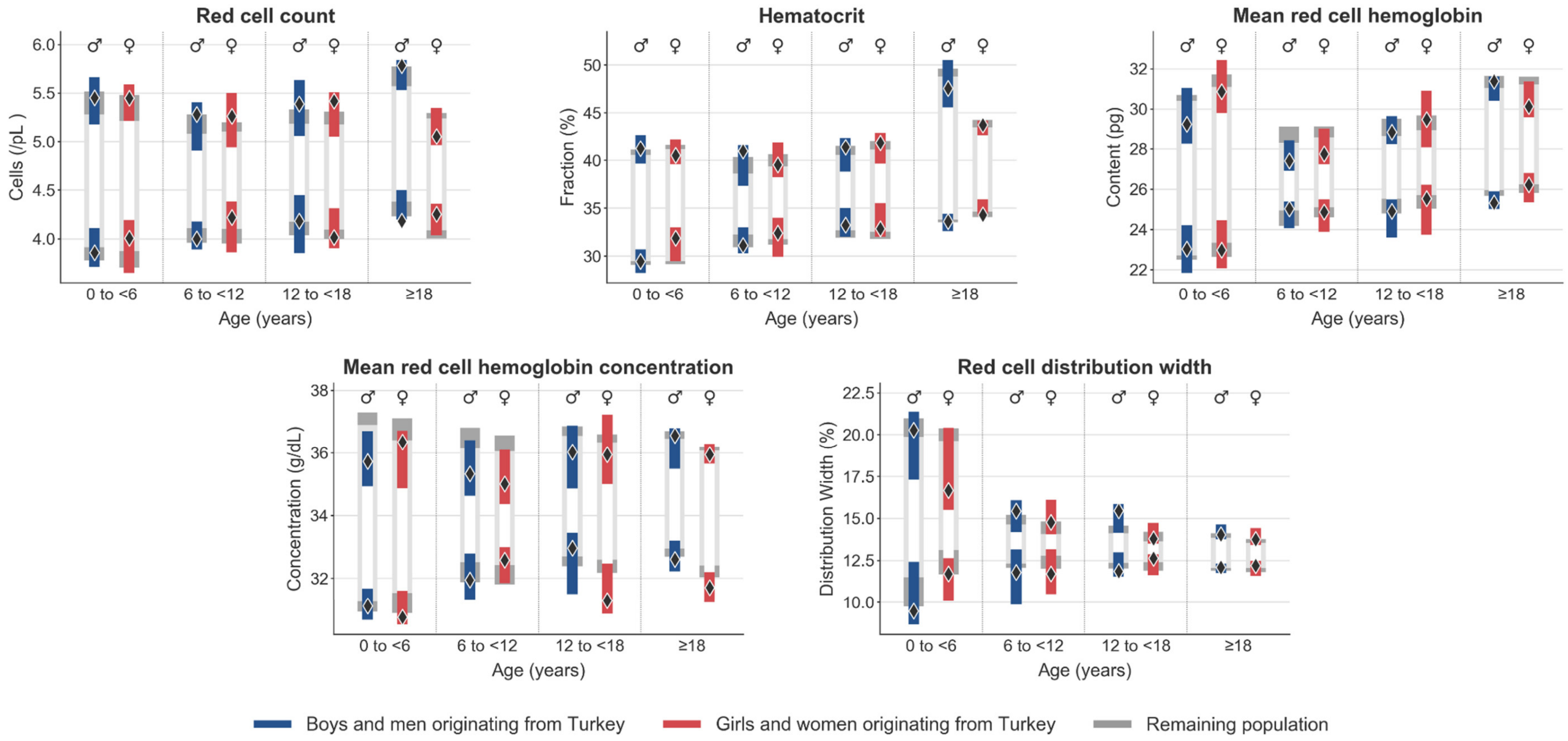

Reference intervals of red cell count, hematocrit, mean red cell hemoglobin, mean red cell hemoglobin concentration, and red cell distribution width for individuals originating from Turkey. Reference intervals and 90 % confidence intervals for individuals originating from Turkey (black diamonds denote male and female reference limits and blue and red bars denote the respective 90 % confidence intervals) in comparison to the remaining population (dark-gray bars in the background denote reference limits' confidence intervals). The exact numerical values of the reference intervals and confidence intervals are available in Supplemental Tables 2 and 3. For hemoglobin, mean corpuscular volume (MCV), platelet count, and white cell count see Figure 2.
